# A 53-Year-Old Man with Intermittent Colicky Abdominal Pain due to *Fasciola* Incarceration in Common Bile Duct: A Case Report

**Published:** 2018

**Authors:** Davood YADEGARINIA, Shabnam TEHRANI, Zahra DOOSTI

**Affiliations:** Infectious Diseases and Tropical Medicine Research Center, Shahid Beheshti University of Medical Sciences, Tehran, Iran

**Keywords:** Fascioliasis, Cholangiocarcinoma, Endoscopic retrograde cholangiopancreatography

## Abstract

Fascioliasis is a zoonotic disease caused by liver flukes of the genus *Fasciola*, as *F. hepatica,* and *F. gigantica*, mainly affecting the liver and biliary system during the chronic phase. These trematodes migrate through biliary ducts results in mild inflammation, when it is difficult to distinguish from obstructive lesions. Here we describe a 53-yr-old man from Golpayegan, a city in Isfahan Province, Iran, in year 2015, with occasional fever and chills, and also frequent colicky abdominal pain mainly on the right upper quadrant, with tenderness at that part. There was no jaundice and elevated bilirubin, but increased alkaline phosphatase was detected. Dilated common bile duct on abdominal sonography, without any visible lesion at its end and also dilated intra- and extrahepatic biliary ducts on abdominal CT-scan were seen. Endoscopic Retrograde Cholangiopancreatography (ERCP) detected incarceration of parasites behind Oddi’s sphincter and also in common bile duct and serologic test (ELISA) confirmed fascioliasis. However, Iran is one of the most affected countries by *Fasciola*, being aware of rare symptoms and presentations of this disease can aid the physicians to make timely and accurate diagnosis and therefore reduce the consequent morbidities.

## Introduction

Fascioliasis is a parasitic zoonosis presenting with right upper quadrant pain, loss of appetite, nausea, diarrhea, respiratory symptoms and urticaria in acute phase, affecting mostly the liver and biliary system late in its course. The parasite is transmitted to humans by ingestion of water-cress, water, and foods contaminated by meta-cercariae. *Fasciola hepatica* and *F. gigantica* are the causal agents of the disease and livestock such as cattle, sheep, goats, and buffalos are the main reservoir hosts. In chronic phase of fascioliasis, marked inflammation resulting in biliary system hyperplasia and biliary lithiasis occurs and although this phase is usually asymptomatic, extrahepatic cholestasis, jaundice, and pruritus may be eventually seen.

Fascioliasis is one of the most important helminthic infections in Iran and WHO has included Iran among six countries having serious problems with the disease (1). Therefore, with regard to the importance of human fascioliasis in Iran, the physicians must be aware of different, even rare presentations of this infection in order to make the most accurate diagnosis and apply the best treatment as early as possible. Among presentations of fascioliasis, although signs of cholestasis and biliary obstruction mimicking cholangiocarcinoma are rare, there are several reports of these presentations from Iran (2, 3) and all over the world (4–8), which *Fasciola* was eventually driven as the causal agent.

Here we report a patient with relapsing colicky abdominal pain, weight loss and symptoms of biliary obstruction without jaundice, finally diagnosed as fascioliasis by ERCP and serologic tests.

## Case presentation

A 53-yr-old man from Golpayegan, Isfahan Province, central Iran, with past history of cholecystectomy referred with complaint of vague colicky epigastric pain begun 2 yr from presentation and had fluctuation during the time. The pain became severe and constant gradually, with the peak about 4 months ago, led him to seek medical attention. There was no relationship between food consumption, especially high fat-containing meals, and pain severity. At the beginning of the disease course, the pain had been relieved by anti-acids but eventually, none of the outpatient medications made him free of pain. He did not mention any urticarial reaction, jaundice, nausea, vomiting, night sweats or pruritus during these two years, but he had history of occasional fever without any specific pattern accompanied by chills within this period. He did not have anorexia, but had increased appetite instead; constipation was a cardinal symptom along with malaise. Significant weight loss (about 10 kg) occurred late in the course of his disease (i.e. the last 2 months).

The patient agreed to be presented in this report and a written consent was signed by him. He was formerly a farmer and husband-man and from one year before the onset of disease (i.e. 3 yr from now), he was a trucker and had history of traveling around the country and taking unsafe and insanitary food and water. He denied consumption of any illicit drugs, alcohol, and opium at all, but he used to smoke cigarettes. No special medication he was taking.

On physical examination, he was afebrile and moderately ill, with stable vital signs and normal sclera without icterus. There was no rash or signs of excoriation, no conjunctivitis, no lymphadenopathy, no gynecomastia and abdominal distention. There was not any abnormality in cardiovascular and lung examination. Right upper quadrant tenderness existed on abdominal palpation, without rebound tenderness and organomegaly. Musculoskeletal and neurologic examinations were normal. The results of initial laboratory investigations are presented in Table 1. The first colonoscopy survey was normal and initial evaluation by upper gastrointestinal endoscopy revealed mild antral gastritis. Abdominal ultrasonography revealed dilated CBD without any visible lesion at its end. A hypo-echo mass lesion suggesting pathologic lymph node was seen lateral to aortic bifurcation, at left side. Abdominal CT-scan suggested dilated intra- and extrahepatic biliary ducts and CBD with 10 mm diameter and also some large intestinal lymph nodes with compressive effect on adjacent structures.

**Table 1:** Initial laboratory investigations results

**Variable**	**Rate**
WBC count: 21.540 ×10^3^/μl (elevated)	BUN: 12 mg/dL (normal)
(Eosinophil: 1.5% of concurrent WBC count)	Serum Cr: 0.7 mg/dL (normal)
Hb: 14.2 g/dL (normal)	Fasting blood sugar: 106 mg/dL
MCV: 84.2 fL (normal)	Sodium: 136 meq/L (normal)
Platelet: 230 ×10^3^/μl (normal)	Potassium: 4.5 meq/L (normal)
total serum bilirubin: 1.2 mg/dL (normal))	Calcium: 8.9 mg/dL (normal)
Direct bilirubin: 0.7 mg/dL (normal)	Phosphorus: 4.1 mg/dL (normal)
Alkaline phosphatase: 684 IU/L (elevated)	Amylase: 62 IU/L (normal)
urine analysis: normal	Lipase: 40 IU/L (normal)
Aspartate Aminotransferase (AST):	105 U/L (elevated
Alanine Aminotransferase (ALT):	70 U/L (elevated)
ESR(1 hour):	14 mm/hr (normal)
CRP:	56 (elevated)
CEA:	1.23 ng/mL (normal)
CA19-9(ECL):	7.2 U/mL (normal)
AFP(ECL):	0.77 IU/mL (normal)

The second upper gastrointestinal endoscopic survey had the same result as the first.

During Endoscopic retrograde cholangiopancreatography (ERCP), dilated common bile duct (CBD) about 12 millimeter and large ampulla were seen. After sphincterotomy, multiple flat whitish parasites were incarcerated behind Oddi’s sphincter and within common bile duct, which extracted. There was no stone in CBD and ampulla did not contain any mass. Serologic exam, using ELISA method was performed by somatic antigen of *Fasciola* and yielded positive according to the reference laboratory cut-off.

The patient was treated with triclabendazole 250 mg, three tablets daily, for two executive days (six tablets totally) successfully and he pains relieved after ERCP and full course of treatment. He advised not to use unsanitary food and water. On follow up, he was well and did not mention any similar abdominal pain as before.

## Discussion

Up to 17 million cases have been estimated to be affected with fascioliasis worldwide (9). There are several articles noticed the heavy socioeconomic burden of infections caused by trematodes including *Fasciola* spp. (10, 11). Despite of them, lack of global holistic burden estimation for food-borne trematodiases exists and multiple articles have been written to emphasize on the importance of these tropical infections (12, 13). Several studies have been done for evaluating the prevalence of human fascioliasis and considering better diagnostic measures around the world (14, 15). Nowadays, because of inappropriate attention to human fascioliasis although it’s heavy global socioeconomic burden, this infection is recognized by WHO as one of the neglected tropical diseases (16).

Iran is one of the countries that fascioliasis is troublesome in them (1). Two outbreaks of fascioliasis in north of Iran occurred up to now, and it is estimated that more than 10000 and 5000 people were affected in the first and second one respectively (17, 18), therefore investigating the burden of these epidemics and searching for seroepidemiology of this infection in Iran were of value among researchers and several articles had been published in this field (19, 20). More prevalence of this disease in north of Iran, especially in Guilan, maybe because of the dietary habits in this region and particular climate (17, 21–23). A study performed over a three-year period from Mar 2008 to Mar 2011 claimed that human fascioliasis is hypoendemic in Guilan Province and recommended a passive case-finding approach and effective veterinary public health measures for control of this infection in humans (18). Therefore, the importance of fascioliasis as an occasionally silent helminthic infection which causes morbidities resulting in frequent medical visits and hospitalizations has become evident during the time.

As we know, *F. hepatica* is one of the liver flukes which migrate through biliary ducts in chronically infected people, results in biliary inflammation which is usually asymptomatic but can occasionally lead to biliary obstruction presenting with the related symptoms and elevated erythrocyte sedimentation rate (ESR), alkaline phosphatase, Gamma-glutamyl transferase and bilirubin. In this phase, it is difficult to distinguish from obstructive lesions, and there are studies revealed different features of that, such as cholangitis (4), sphincter of Oddi Dysfunction (5), recurrent biliary colics (6), common bile duct (CBD) tumors and cholangiocarcinoma (2, 3, 7, 8). These presentations are because of excessive inflammation within biliary structures which results in misdiagnosis.

Similar to ours, there are several reports of fascioliasis, which the patients presented with abnormal presentations in them. For example, an elderly man from Iran was described, who had Jaundice and obstruction in biliary system and finally he was diagnosed as fascioliasis (2). Moreover, an elderly woman was reported with the history of contaminated water and vegetable consumption, who had features of cholangitis and during ERCP, three living *F. hepatica* was extracted from her biliary tract (3). In another report, a young man was described with four years of recurrent biliary colics, in whom *Fasciola* was discovered in his biliary tree during ERCP (6). In our patient according to abdominal colics, dilated common bile duct (CBD) and elevated alkaline phosphatase, the initial impression was cholangiocarcinoma; but lack of hyperbilirubinemia and even pruritus was a question in him. Regarding the history of unsafe water and vegetable consumption, and also the presence of dilated CBD, the suspicion for fascioliasis fortified, and despite he did not have eosinophilia, serologic evaluation was done. ERCP as a diagnostic method for possible diagnoses performed, but it was also therapeutic in him.

## Conclusion

With regard to this report and other ones presenting rare signs and symptoms of this infection and also the history of fascioliasis in Iran, it is essential to keep these various features in mind, in order to apply the most appropriate diagnostic test for this infection beside work-ups for other differential diagnoses. As we know, fascioliasis is an easy to treat infection and making the diagnosis and recommending the best treatment and medical procedure at early stages, can easily prevent poor outcomes, stigmas, and complications.

## References

[1] Ashrafi K. The Status of Human and Animal Fascioliasis in Iran: A Narrative Review Article. Iran J Parasitol. 2015; 10(3):306–28. 26622287 PMC4662732

[2] Moghadami M Mardani M. *Fasciola hepatica*: A cause of obstructive Jaundice in an elderly man from Iran. Saudi J Gastroenterol. 2008; 14(4):208–10. 19568541 10.4103/1319-3767.43279 PMC2702927

[3] Niknam R Kazemi MH Mahmoudi L. Three Living *Fasciola Hepatica* in the Biliary Tract of a Woman. Iran J Med Sci. 2015; 40(5):465–8. 26379355 PMC4567608

[4] Ha JS Choi HJ1 Moon JH Lee YN Tae JW Endoscopic Extraction of Biliary Fascioliasis Diagnosed Using Intraductal Ultrasonography in a Patient with Acute Cholangitis. Clin Endosc. 2015; 48(6):579–82. 26668810 10.5946/ce.2015.48.6.579 PMC4676668

[5] Keshishian J Brantley SG Brady PG. Biliary fascioliasis mimicking sphincter of Oddi dysfunction. South Med J. 2010; 103(4):366–8. 20224481 10.1097/SMJ.0b013e3181d413d8

[6] Al Qurashi H Masoodi I Al Sofiyani M Al Musharaf H Shaqhan M All GN. Biliary fascioliasis - an uncommon cause of recurrent biliary colics: Report of a case and brief review. Ger Med Sci. 2012; 10:Doc10. 22566787 10.3205/000161 PMC3342511

[7] Losada H Hirsch M Guzmán P Fonseca F Hofmann E Alanís M. Fascioliasis simulating an intrahepatic cholangiocarcinoma - Case report with imaging and pathology correlation. Hepatobiliary Surg Nutr. 2015; 4(1):E1–7. 25713810 10.3978/j.issn.2304-3881.2014.09.15 PMC4318963

[8] Alshekhani M Hussein H Karbuli T Kasnazan K. Large number of *Fasciola hepatica* discovered during endoscopic retrograde cholangiopancreatography (ERCP). Case Rep Clin Med. 2013; 2(2):177–78.

[9] Fürst T Keiser J Utzinger J. Global burden of human food-borne trematodiasis: a systematic review and meta-analysis. Lancet Infect Dis. 2012; 12(3):210–21. 22108757 10.1016/S1473-3099(11)70294-8

[10] Torgerson PR Macpherson CN. The socioeconomic burden of parasitic zoonoses: Global trends. Vet Parasitol. 2011; 182(1):79–95. 21862222 10.1016/j.vetpar.2011.07.017

[11] Nyindo M Lukambagire AH. Fascioliasis: An Ongoing Zoonotic Trematode Infection. Biomed Res Int. 2015; 2015:786195. 26417603 10.1155/2015/786195 PMC4568335

[12] Hotez PJ Brindley PJ Bethony JM King CH Pearce EJ Jacobson J. Helminth infections: the great neglected tropical diseases. J Clin Invest. 2008; 118(4):1311–21. 18382743 10.1172/JCI34261 PMC2276811

[13] Huppatz C Durrheim DN. Control of Neglected Tropical Diseases. N Engl J Med. 2007; 357(23):2407. 18057348 10.1056/NEJMc072881

[14] Boşnak VK Karaoğlan İ Sahin HH Namiduru M Pehlivan M Okan V Mete AÖ Evaluation of patients diagnosed with fascioliasis: A six-year experience at a university hospital in Turkey. J Infect Dev Ctries. 2016; 10(4):389–94. 27131001 10.3855/jidc.6681

[15] Mekky MA Tolba M Abdel-Malek MO Abbas WA Zidan M. Human fascioliasis: a reemerging disease in upper Egypt. Am J Trop Med Hyg. 2015; 93(1):76–9. 25870421 10.4269/ajtmh.15-0030 PMC4497909

[16] World Health Organization (2010). Working to overcome the global impact of neglected tropical diseases: first WHO report on neglected tropical diseases.

[17] Salahi-Moghaddam A Arfaa F. Epidemiology of Human Fascioliasis Outbreaks in Iran. J Arch Mil Med. 2013; 1(1):6–12.

[18] Ashrafi K1 Saadat F O’Neill S Rahmati B1 Amin Tahmasbi H Pius Dalton J The Endemicity of Human Fascioliasis in Guilan Province, Northern Iran: the Baseline for Implementation of Control Strategies. Iran J Public Health. 2015; 44(4):501–11. 26056669 PMC4441963

[19] Manouchehri Naeini K Mohammad Nasiri F Rokni MB Kheiri S. Seroprevalence of Human Fascioliasis in Chaharmahal and Bakhtiyari Province, Southwestern Iran. Iran J Public Health. 2016; 45(6):774–80. 27648421 PMC5026833

[20] Saberinasab M Mohebali M Molawi G Beigom Kia E Aryaeipour M Rokni MB. Seroprevalence of human fascioliasis using indirect ELISA in Isfahan district, central Iran in 2013. Iran J Parasitol. 2014; 9(4):461–5. 25759726 PMC4345084

[21] Ashrafi K Valero MA Massoud J Sobhani A Solaymani-Mohammadi S Plant-borne human contamination by fascioliasis. Am J Trop Med Hyg. 2006; 75(2):295–302. 16896136

[22] Ashrafi K Valero MA Forghan-Parast K Potential transmission of human fascioliasis through traditional local foods in northern Iran. Iran J Public Health. 2006, 35(2): 57–63.

[23] Halimi M Farajzadeh M Delavari M Arbabi M. Developing a climate-based risk map of fascioliasis outbreaks in Iran. J Infect Public Health. 2015; 8(5):481–6. 25982974 10.1016/j.jiph.2015.04.024

